# Pathological spectrum of glomerular disease in patients with renal insufficiency: a single-center study in Northeastern China

**DOI:** 10.1080/0886022X.2019.1620774

**Published:** 2019-06-14

**Authors:** Liangmei Chen, Manyu Luo, Changqing Dong, Bing Li, Weiguang Zhang, Ping Nie, Juan Liu, Xiangmei Chen, Ping Luo

**Affiliations:** a Department of Nephrology, The Second Hospital of Jilin University, Changchun, People’s Republic of China;; b Department of Nephrology, Chinese PLA General Hospital, Chinese PLA Institute of Nephrology, State Key Laboratory of Kidney Diseases, National Clinical Research Center of Kidney Diseases, Beijing Key Laboratory of Kidney Disease, Beijing, People’s Republic of China

**Keywords:** Glomerular disease, pathology, renal biopsy, renal insufficiency

## Abstract

**Background:** To investigate the pathological spectrum of glomerular disease in patients with renal insufficiency (RI) from 2008 to 2017.

**Methods and results:** We calculated the estimated glomerular filtration rate (eGFR) with the Chronic Kidney Disease Epidemiology Collaboration creatinine (CKD-EPI) equation and defined RI as an eGFR <60 ml/min/1.73 m^2^. A total of 969 RI patients were included in our study. IgA nephropathy (IgAN) was the most common subtype of primary glomerulonephritis (37.2%). The frequencies of IgAN and non-IgA mesangioproliferative glomerulonephritis decreased from 27.3% and 9.5% during 2008–2012 to 20.7% and 2.6% during 2013–2017, respectively. However, the frequency of membranous nephropathy increased from 6.8% to 16.2%. Lupus nephritis was the most common subtype of secondary glomerulonephritis (32.1%). The frequencies of both ANCA-associated systemic vasculitis and diabetic nephropathy increased from 3.8% to 7.6% and from 4.3% to 7.6%, respectively. The number of elderly patients (≥60 years) in our study increased sharply, from 15.6% in 2008 to 35.0% in 2017. Membranous nephropathy, minimal change disease, membranoproliferative glomerulonephritis, lupus nephritis and renal amyloidosis are more frequently observed in the elderly patients than in nonelderly patients (<60 years) (*p* < .05). Excluding those with acute kidney injury, IgAN was the leading cause of RI (24.9%), followed by membranous nephropathy (13.3%) and lupus nephritis (12.0%).

**Conclusions:** IgAN and lupus nephritis were the most prevalent primary glomerulonephritis and secondary glomerulonephritis in patients with RI, respectively. The frequencies of membranous nephropathy, ANCA-associated systemic vasculitis and diabetic nephropathy increased significantly. The number of elderly patients with RI increased sharply.

## Introduction

Chronic kidney disease (CKD) has become a worldwide public health issue due to its high incidence, poor prognosis and extensive economic burden. The prevalence of CKD is 10.8% among the 13 Chinese provinces and is as high as 14.2% in some central cities [1]. Renal insufficiency (RI), as a transitional phase of the progression of CKD to renal failure, requires a precise diagnosis and timely treatment. In the past, percutaneous renal biopsy (PRB) was restricted and even regarded as a contraindication in patients with RI due to the increased risk of complications such as hemorrhage, infection or even death. With improvements in biopsy equipment and techniques, an increasing number of patients with RI have selected PRB, which provides more accurate renal pathology results. In our study, we retrospectively investigated the pathological spectrum of glomerular disease in 969 patients with RI.

## Materials and methods

The clinical and pathological data of RI patients who underwent renal biopsy in the Second Hospital of Jilin University from January 2008 to December 2017 were retrospectively analyzed, excluding those with incomplete records, those younger than 15 years old, those with inadequate biopsies (insufficient glomeruli, less than 10 glomeruli per section), those with ambiguous diagnosis and those with repeated renal biopsy with an unchanged diagnosis.

Patients with an estimated glomerular filtration rate (eGFR)<60 ml/min/1.73 m^2^ were defined as RI patients, and the eGFR was calculated by the Chronic Kidney Disease Epidemiology Collaboration creatinine (CKD-EPI) equation. RI patients [except for those with acute kidney injury (AKI)] were divided into three groups according to the GFR categories in CKD: CKD3 (GFR 30–59 ml/min/1.73 m^2^), CKD4 (GFR 15–29 ml/min/1.73 m^2^) and CKD5 (GFR <15 ml/min/1.73 m^2^) [2]. The patients were divided into three age stratifications for the analysis: 15–49 years (young adult group), 50–59 years (middle-age group), and ≥60 years (elderly group). Patients in the young adult and middle-age groups were described as the nonelderly group for later analyses.

Indications for renal biopsy were categorized into the following clinical syndromes [3,4]: (1) nephrotic syndrome (NS): proteinuria >3.5 g/24 h, with hypoalbuminemia (serum albumin <35 g/L), with or without hematuria; (2) proteinuria (without NS): proteinuria <3.5 g/day or proteinuria >3.5 g/day without hypoalbuminemia, without hematuria or a significant decline in renal function; (3) proteinuria and hematuria: proteinuria plus hematuria, without impaired renal function; (4) AKI: the serum creatinine (SCr) level was doubled in patients with normal renal function, or increased 50% over one year in patients with CKD or SCr >2 mg/dl with unknown baseline renal function; and (5) progressive CKD: SCr increased but less than 50% over one year in patients with CKD. Indications remained unchanged from 2008–2017; thus, we selected cases with RI from these years for our retrospective analysis.

The preferred site for renal biopsy was the lateral aspect of the lower pole of the left kidney under real-time ultrasound guidance with the patient in the prone position. An automated biopsy gun and a 16- or 18-gauge needle were used to ensure that the biopsy sample contained a minimum of 10 glomeruli.

Pathologic diagnosis was made on the basis of the detailed histologic features, which included the results of light microscopy (LM), electron microscopy (EM) and immunofluorescence (IF). LM (hematoxylin and eosin, periodic acid-Schiff, Jones and Masson’s trichrome stains) and IF (for IgG, IgM, IgA, C3, C4, C1q, fibrinogen, albumin, kappa and lambda light chains) were routinely performed for pathological examination of the biopsy specimens on all patients. All antibodies used for immunofluorescence were purchased from Dako (Dako A/S, Glostrup, Denmark). Some patients were suggested to send specimens to the First Hospital of Peking University for EM according to diagnostic needs before November 2014; we began to perform EM in our center afterwards. The percentage of patients who received EM was 48.6% in total.

The histologic findings were classified according to the Revised Protocol for Histological Typing of Glomerulopathy [5] and refer to previous research [3,4,6]. Glomerular disease was divided into primary glomerulonephritis (PGN) and secondary glomerulonephritis (SGN). PGN was classified as follows: IgA nephropathy (IgAN), membranous nephropathy (MN), minimal change disease (MCD), non-IgA mesangioproliferative glomerulonephritis (MsPGN), focal segmental glomerulosclerosis (FSGS), membranoproliferative glomerulonephritis (MPGN), crescentic glomerulonephritis (CreGN, defined as CreGN not fulfilling the criteria for systemic disease), and endocapillary proliferative glomerulonephritis (EnPGN). SGN was classified into four categories according to the primary disease: vascular disease, including anti-neutrophil cytoplasmic antibodies (ANCA)-associated systemic vasculitis (AASV), malignant nephrosclerosis (MANS), benign arteriolar nephrosclerosis (BANS), thrombotic microangiopathy (TMA); systemic disease, including lupus nephritis (LN), Henoch–Schönlein purpura nephritis (HSPN); metabolic disease, including diabetic nephropathy (DN), light-chain deposition nephropathy (LCDN), renal amyloidosis (RAMY) and HBV-associated nephritis (HBVN) as infectious disease.

### Statistical analysis

Data were analyzed using SPSS 12.0 (SPSS, Chicago, USA), and graphs were constructed using GraphPad Prism 6 (GraphPad, La Jolla, USA) for Mac. The cases frequencies are expressed as percentages, and numerical variables in the descriptive analyses are reported as the means ± SDs or medians as appropriate for their distribution. The chi-square test or Fisher exact test was used for categorical variables to test differences in disease frequencies between different groups, and the Mann–Whitney *U* test was used for non-normally distributed parameters. Differences were considered statistically significant if *p* < .05.

## Results

### Demographic and clinical characteristics of RI patients diagnosed with glomerular disease

From January 2008 to December 2017, a total of 969 RI patients (534 males and 435 females) were diagnosed with glomerular disease. The median eGFR level was 37 ml/min/1.73 m^2^, and the median age was 48 years old. Approximately 52.3% of patients were in the young adult group (15–49 years), and the percentage of elderly patients was 26.6%, which was higher than that of the middle-age group (21.1%). The number of patients increased from 149 during 2008–2009 to 252 during 2016–2017. The median eGFR and median age were not significantly different between males and females (*p* = .551, *p* = .231). The distributions of age stratification and the time of receiving renal biopsies were not significantly different between different sexes, as shown in Table 1 (*p* = .154, *p* = .405).

**Table 1.** Demographic and clinical characteristics of patients with renal insufficiency.

Characteristics	Total, *n* = 969	Sex		*p*
		Male, *n* = 534	Female, *n* = 435	
eGFR, ml/min/1.73 m^2^, median	37	39	36	.551
Age, years, median	48	50	47	.231
Age stratification, *n* (%)				.154
15–49	507 (52.3)	265 (49.6)	242 (55.6)	
50–59	204 (21.1)	116 (21.7)	88 (20.2)	
≥60	258 (26.6)	153 (28.7)	105 (24.1)	
Time of kidney biopsy, *n* (%)				.405
2008–2009	149 (15.4)	72 (13.5)	77 (17.7)	
2010–2011	169 (17.4)	100 (18.7)	69 (15.9)	
2012–2013	167 (17.2)	93 (17.4)	74 (17.0)	
2014–2015	232 (23.9)	130 (24.3)	102 (23.4)	
2016–2017	252 (26.0)	139 (26.0)	113 (26.0)	

### Pathological distribution of glomerular disease in patients with RI

As shown in Table 2, PGN was the most common glomerular disease in patients with RI, accounting for 63.1% of cases. SGN accounted for 36.9%, and the proportions of glomerular disease caused by vascular disease, systemic disease, metabolic disease and infectious disease were 14.1%, 13.6%, 8.3%, and 0.9% respectively.

**Table 2.** Spectrum of glomerular disease in patients with renal insufficiency.

Pathology type of glomerular disease	Number	Percentage
Primary glomerulonephritis	611	63.1%
IgA nephropathy	227	37.2%
Membranous nephropathy	119	19.5%
Minimal change disease	70	11.5%
Non-IgA mesangioproliferative glomerulonephritis	53	8.7%
Focal segmental glomerulosclerosis	74	12.1%
Membranoproliferative glomerulonephritis	29	4.7%
Crescentic glomerulonephritis	27	4.4%
Endocapillary proliferative glomerulonephritis	12	2.0%
Secondary glomerulonephritis	358	36.9%
Lupus nephritis	115	32.1%
Henoch–Schönlein purpura nephritis	17	4.7%
ANCA-associated systemic vasculitis	58	16.2%
Malignant nephrosclerosis	39	10.9%
Benign arteriolar nephrosclerosis	38	10.6%
Thrombotic microangiopathy	2	0.6%
Diabetic nephropathy	60	16.8%
Light-chain deposition nephropathy	8	2.2%
Renal amyloidosis	12	3.4%
HBV-associated nephritis	9	2.5%

ANCA: anti-neutrophil cytoplasmic antibodies; HBV: hepatitis B virus.

IgAN was the most common subtype of PGN, accounting for 37.2%. Patients with MN and FSGS accounted for 19.5% and 12.1% of cases in PGN, respectively. The percentages of MCD and MsPGN were 11.5% and 8.7%, respectively. All remaining subtypes of PGN had a percentage of no more than 5%.

LN was the most frequently observed SGN, with a percentage of 32.1%, followed by DN (16.8%) and AASV (16.2%). MANS and BANS was found in 39 and 38 patients, respectively, accounting for 10.9% and 10.6%, respectively. All remaining subtypes of SGN had percentages of less than 5%.

### Changing frequencies of glomerular diseases in patients with RI during the last 10 years

To demonstrate how the pathological spectrum changed during the last 10 years, we calculated the cases of each pathological subtype during 2008–2012 and 2013–2017 separately and analyzed the frequency of glomerular diseases at different times, as shown in Table 3.

**Table 3.** Comparison of the frequencies of glomerular disease in patients with renal insufficiency at different times.

Pathology type of glomerular disease	2008–2012	2013–2017	*p*
Primary glomerulonephritis, *n* (%)			
IgA nephropathy	109 (27.3)	118 (20.7)	**.018**
Membranous nephropathy	27 (6.8)	92 (16.2)	**<.001**
Minimal change disease	22 (5.5)	48 (8.4)	.082
Non-IgA mesangioproliferative glomerulonephritis	38 (9.5)	15 (2.6)	**<.001**
Focal segmental glomerulosclerosis	32 (8.0)	42 (7.4)	.721
Membranoproliferative glomerulonephritis	16 (4.0)	13 (2.3)	.123
Crescentic glomerulonephritis	13 (3.3)	14 (2.5)	.462
Endocapillary proliferative glomerulonephritis	3 (0.8)	9 (1.6)	.391
Secondary glomerulonephritis, *n* (%)			
Lupus nephritis	56 (14.0)	59 (10.4)	.085
Henoch–Schönlein purpura nephritis	9 (2.3)	8 (1.4)	.324
ANCA-associated systemic vasculitis	15 (3.8)	43 (7.6)	**.014**
Malignant nephrosclerosis	17 (4.3)	22 (3.9)	.765
Benign arteriolar nephrosclerosis	13 (3.3)	25 (4.4)	.367
Thrombotic microangiopathy	1 (0.3)	1 (0.2)	1.000
Diabetic nephropathy	17 (4.3)	43 (7.6)	**.035**
Light-chain deposition nephropathy	2 (0.5)	6 (1.1)	.563
Renal amyloidosis	5 (1.3)	7 (1.2)	1.000
HBV-associated nephritis	5 (1.3)	4 (0.7)	.593

ANCA: anti-neutrophil cytoplasmic antibodies; HBV: hepatitis B virus. Data shown on bold are *p* < 0.05.

In PGN, the frequencies of IgAN, MN and MsPGN changed significantly. The frequencies of IgAN and MsPGN declined from 27.3% and 9.5% during 2008–2012 to 20.7% and 2.6% during 2013–2017, respectively. However, the frequency of MN increased from 6.8% to 16.2%. No significant changes were observed in the frequencies of other types of PGN.

In SGN, the frequencies of AASV and DN were both increased, with the former increasing from 3.8% to 7.6% and the latter increasing from 4.3% to 7.6%. No significant changes were observed in the frequencies of other SGN.

### The pathological spectrum of glomerular diseases in elderly patients with RI

**Figure 1.**
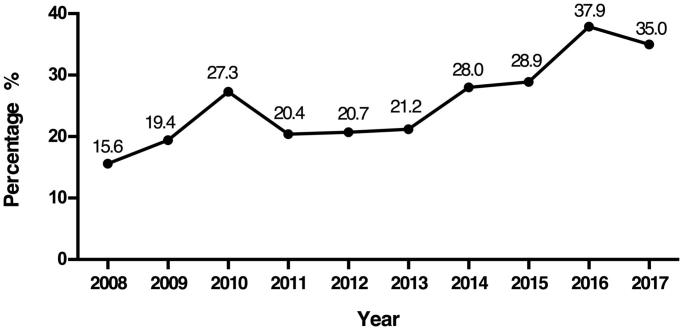
Percentage of elderly patients (≥60 years) during the period from 2008 to 2017.

In RI patients diagnosed with glomerular disease, the number of elderly patients markedly increased. The percentage of elderly patients increased from 15.6% in 2008 to 35.0% in 2017 (Figure 1). We then compared the distributions of glomerular diseases in the elderly group and nonelderly group, as shown in Table 4. In PGN, the proportions of MN, MCD and MPGN in the elderly group were higher than those in the nonelderly group, while the prevalence of IgAN was lower in the elderly group than in the nonelderly group. In SGN, the proportions of LN and renal amyloidosis in the elderly group were higher than those in the nonelderly group, while the prevalence rates of HSPN and BANS were lower in the elderly group than in the nonelderly group.

**Table 4.** Comparison of glomerular disease distribution in the elderly and nonelderly groups.

Pathology type of glomerular disease	Elderly group	Nonelderly group	*p*
Primary glomerulonephritis, *n* (%)			
IgA nephropathy	20 (11.6)	207 (47.3)	**<.001**
Membranous nephropathy	66 (38.2)	53 (12.1)	**<.001**
Minimal change disease	29 (16.8)	41 (9.4)	**.010**
Non-IgA mesangioproliferative glomerulonephritis	15 (8.7)	38 (8.7)	.998
Focal segmental glomerulosclerosis	16 (9.2)	58 (13.2)	.173
Membranoproliferative glomerulonephritis	13 (7.5)	16 (3.7)	**.043**
Crescentic glomerulonephritis	10 (5.8)	17 (3.9)	.303
Endocapillary proliferative glomerulonephritis	4 (2.3)	8 (1.8)	.697
Secondary glomerulonephritis, *n* (%)			
Lupus nephritis	27 (31.8)	31 (11.4)	**<.001**
Henoch–Schönlein purpura nephritis	1 (1.2)	38 (13.9)	**.001**
ANCA-associated systemic vasculitis	9 (10.6)	29 (10.6)	.993
Malignant nephrosclerosis	2 (2.4)	0 (0.0)	.088
Benign arteriolar nephrosclerosis	10 (11.8)	105 (38.5)	**<.001**
Thrombotic microangiopathy	4 (4.7)	13 (4.8)	.983
Diabetic nephropathy	19 (22.4)	41 (15.0)	.114
Light-chain deposition nephropathy	3 (3.5)	5 (1.8)	.614
Renal amyloidosis	8 (9.4)	4 (1.5)	**.001**
HBV-associated nephritis	2 (2.4)	7 (2.6)	1.000

ANCA: anti-neutrophil cytoplasmic antibodies; HBV: hepatitis B virus. Data shown on bold are *p* < 0.05.

### Distribution of glomerular disease among patients with different GFR categories

Seventy-one RI patients with glomerular disease were diagnosed with AKI; among these patients, 38.0% had AASV, 22.5% had MANS, and 18.3% had CreGN. After excluding patients with AKI, we analyzed the pathological distribution of CKD patients with different GFR categories (CKD3-5), as shown in Table 5. Overall, IgAN was the leading cause of RI to different levels of GFR (24.9%), followed by MN (13.3%) and LN (12.0%). The difference in the proportional distribution of MN, CreGN, AASV and MANS in different groups was significant (*p* < .01). CreGN, AASV and MANS patients had higher percentages in CKD5 than in CKD3 (*p* < .01). Approximately 78.6% of CreGN patients and nearly 47.8% of MANS patients had GFR <15 ml/min/1.73 m^2^. In AASV, 41.9% of the patients had GFR of 15–29 ml/min/1.73 m^2^. Approximately 76.5% of MN patients had GFR of 30–59 ml/min/1.73 m^2^.

**Table 5.** Glomerular disease spectrum of patients with varying degrees of renal insufficiency.

Pathology type	CKD5	CKD4	CKD3	Total	*p*
Primary glomerulonephritis, *n* (%)					
IgA nephropathy	28 (12.5)	41 (18.3)	155 (69.2)	224 (24.9)	.338
Membranous nephropathy	6 (5.0)	22 (18.5)	91 (76.5)*	119 (13.3)	**.014**
Minimal change disease	5 (7.4)	20 (29.4)	43 (63.2)	68 (7.6)	.185
Non-IgA mesangioproliferative glomerulonephritis	5 (9.4)	9 (17.0)	39 (73.6)	53 (5.9)	.499
Focal segmental glomerulosclerosis	6 (8.2)	11 (15.1)	56 (76.7)	73 (8.1)	.138
Membranoproliferative glomerulonephritis	5 (17.2)	3 (10.3)	21 (72.4)	29 (3.2)	.218
Crescentic glomerulonephritis	11 (78.6)	2 (14.3)*	1 (7.1)*	14 (1.6)	**<.001**
Endocapillary proliferative glomerulonephritis	0 (0.0)	0 (0.0)	11 (100)	11 (1.2)	.081
Secondary glomerulonephritis, *n* (%)					
ANCA-associated systemic vasculitis	10 (32.3)	13 (41.9)	8 (25.8)*#	31 (3.5)	**<.001**
Malignant nephrosclerosis	11 (47.8)	3 (13.0)*	9 (39.1)*	23 (2.6)	**<.001**
Benign arteriolar nephrosclerosis	2 (5.4)	10 (27.0)	25 (67.6)	37 (4.1)	.415
Thrombotic microangiopathy	0 (0.0)	2 (100)	0 (0.0)	2 (0.2)	.062
Lupus nephritis	12 (11.1)	30 (27.8)	66 (61.1)	108 (12.0)	.289
Henoch–Schönlein purpura nephritis	1 (5.9)	3 (17.7)	13 (76.5)	17 (1.9)	.668
Diabetic nephropathy	5 (8.3)	20 (33.3)	35 (58.3)	60 (6.7)	.074
Light-chain deposition nephropathy	1 (12.5)	2 (25.0)	5 (62.5)	8 (0.9)	.876
Renal amyloidosis	0 (0.0)	4 (33.3)	8 (66.7)	12 (1.3)	.401
HBV-associated nephritis	0 (0.0)	1 (11.1)	8 (88.9)	9 (1.0)	.524

ANCA: anti-neutrophil cytoplasmic antibodies; HBV: hepatitis B virus; CKD3: GFR 30–59 ml/min/1.73 m^2^; CKD4: GFR 15–29 ml/min/1.73 m^2^; CKD5: GFR < 15 ml/min/1.73 m^2^. Data shown on bold are *p* < 0.05.

* *p* < .05 for comparison with CKD5.

# *p* < .05 for comparison with CKD4.

**Figure 2.**
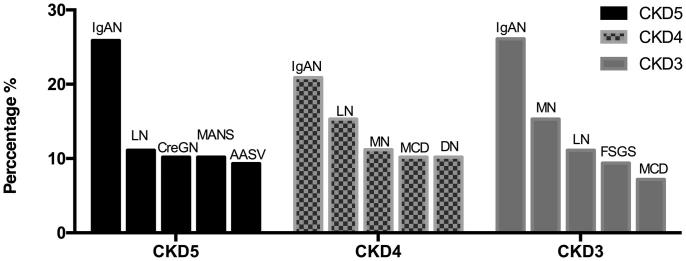
Five most frequently observed glomerular diseases among patients with different degrees of renal insufficiency. CKD3: GFR30-59 ml/min/1.73 m^2^; CKD4: GFR 15–29 ml/min/1.73 m^2^; CKD5: GFR < 15 ml/min/1.73 m^2^. IgAN: IgA nephropathy; LN: lupus nephritis; CreGN: crescentic glomerulonephritis; MANS: malignant nephrosclerosis; AASV: anti-neutrophil cytoplasmic antibodies associated systemic vasculitis; MN: membranous nephropathy; MCD: minimal change disease; DN: diabetic nephropathy; FSGS: focal segmental glomerulosclerosis.

Figure 2 shows the five most frequently observed glomerular diseases in each group. IgAN was the dominant disease in the CKD3-5 groups. LN was also found to be prevalent in these three groups. CreGN, AASV and MANS were the primary diseases in the CKD5 group. MN was observed in 11.2% patients in the CKD4 group and in 26.1% patients in the CKD3 group.

## Discussion

Due to improvements in technique, PRBs are widely performed in RI patients, with few complications reported. This study was the first to present a kidney biopsy series of RI patients with glomerular disease in northeastern China and to provide detailed information on the distributions of biopsy-proven glomerular diseases, which vary among patients with different degrees of RI.

In terms of sex composition, the sex ratio (male: female) of glomerular disease patients with RI was 1.23:1. The sex ratios of data in all renal biopsies from the United States, Brazil and central China were 0.90:1, 0.96:1 and 0.87:1, respectively [7–9]. However, in Wales and northern Germany, the ratio was 1.70:1 [10,11]. Animal studies have shown that renal impairment is associated with androgen secretion. Male hormones may aggravate renal alterations, but this conjecture requires further research and verification in humans [12]. Young adult patients (15–49 years) were the most prevalent patients with glomerular disease in our study, accounting for 52.3%. The proportion of elderly patients (≥60 years) in our study was 26.6%, which is much higher than the percentage of the elderly patients among all renal biopsy patients reported by Shanghai Tongji Hospital (17.1%) [13]. Elderly individuals are more susceptible to kidney damage because glomeruli become sclerotic and the glomerular filtration rate declines with age. The mechanism is related to hemodynamic changes and vascular lesions in the glomeruli. The aging of China’s population is problematic, and the renal pathological distribution in elderly patients is receiving increasing attention. An increasing tendency of elderly patients receiving renal biopsies was observed in our study, which may contribute to the pathological spectrum of age-associated glomerular disease.

PGN was the major cause of glomerular disease (63.1%), which is consistent with previous reports [9,14,15]. The subtypes of PGN with the highest proportion in our study were IgAN (37.2%), followed by MN (19.5%). Epidemiological studies have shown a marked increase in the prevalence of MN in multiple regions of China [9,16,17], and the percentage of MN also increased significantly in our study (*p* < .001). This result is consistent with that from Peking University First Hospital [18]. Some researchers found that the exposure of particulate matter 2.5 (PM_2.5_) may be associated with an increased risk of MN in China, and this relationship is nonlinear. For PM_2.5_ > 70 µg/m^3^, each 10 µg/m^3^ increase in PM_2.5_ concentration is associated with a 14% greater risk of developing MN [6]. Northeast China is a heavily industrialized area with severe air pollution, which could explain the increasing number of MN patients in our medical center during these years [19]. The pathological distribution of glomerular disease varies by region, race and age. IgAN is also the most common glomerular disease in Asia and Europe and among American Caucasians [17,20,21], but FSGS is more common among African Americans [22]. FSGS is predominant in North America, while IgAN and FSGS are predominant in Latin America [23]. The percentages of IgAN and MsPGN were found to decrease significantly in our study. A study from South Korea showed that the relative frequency of MsPGN decreased while the relative frequency of IgAN increased in 1992–2011 [24].

LN is the main pathological type of SGN in the Brazil, Cyprus and the Czech Republic [8,25,26]. Among glomerular diseases caused by systemic and metabolic diseases, we observed high prevalence rates of LN and DN. DN is the leading cause of end-stage kidney disease (ESKD) in developed countries [27], and studies have also shown that DN has exceeded PGN as the predominant cause of CKD in China [28]. In our center, the proportion of patients with DN also increased from 4.3% during 2008–2009 to 7.6% during 2016–2017. In clinical practice, nondiabetic kidney disease is easily misdiagnosed as diabetic kidney disease; thus, diabetic mellitus patients with RI should undergo PRB so that the correct diagnosis is made [29]. We observed increasing trends in the frequencies of AASV and vascular disease. RI caused by hypertension may be associated with a high-salt, high-fat diet as well as the geographic location of our center [30]. In Australia, renovascular disease was the most common renal disease (38.4%), and the proportion was as high as 63% in patients >65 years old [31]. Blood pressure management cannot be ignored in RI patients.

We compared the distributions of glomerular disease in elderly and nonelderly patients, and the results showed that the elderly patients with MN, MCD, MsPGN, LN and RAMY were more likely to develop into RI. However, the results for elderly patients with IgAN and BANS were contradictory. As China’s aging problem continues to worsen, it is essential for nephrologists to determine how to prevent aging-related renal disease.

The glomerular disease spectrum of RI patients was analyzed in our research excluding AKI. IgAN was the leading cause in RI of different degrees due to its high incidence. Most glomerular diseases cause only mild RI, except for crescentic glomerulonephritis, AASV, thrombotic microangiopathy and MANS. These diseases generally have more serious pathological damage; thus, this result was considered reasonable.

PRB is still the gold standard for diagnosing glomerular disease in RI patients. Our study helps to elucidate the pathological distribution of RI patients in northeastern China. We believe that these epidemiological data may provide useful information for analyzing the disease states leading to RI and renal failure. There are also several limitations in our study. First, patients may have multiple glomerular diseases at the same time. For example, diabetic patients can have both diabetic kidney disease and nondiabetic kidney disease [32]. The final diagnosis in our study was the most important and obvious one, and each diagnosis was not regarded as a separate observation if two or more diagnoses were identified from a single-biopsy specimen. Second, due to the 48.6% usage of EM, we may have misdiagnosed some specific kinds of kidney disease in which EM is especially essential, as this lack may have affected the accuracy of the diagnoses to some extent. Third, there have been some changes in procedure availability, the maturity of renal biopsy, the proficiency of doctors in kidney biopsy and the levels of understanding among patients during the last ten years. These factors may be potential limitations in this study.

In conclusion, the pathological spectrum of glomerular disease patients with RI changed dramatically, especially for MN, IgAN, MsPGN, AASV and DN. The number of elderly patients with glomerular disease increased sharply over the study period. Renal biopsy is important for patients with RI for early diagnosis and timely treatment.
